# Neighborhood-level social determinants of health burden among adolescent and young adult cancer patients and impact on overall survival

**DOI:** 10.1093/jncics/pkae062

**Published:** 2024-07-25

**Authors:** Elizabeth R Rodriguez, Tori Tonn, Midhat Jafry, Sairah Ahmed, Branko Cuglievan, J Andrew Livingston, Christopher R Flowers, Gregory J Aune, Karen H Albritton, Michael E Roth, Qian Xiao, Michelle A T Hildebrandt

**Affiliations:** Department of Lymphoma/Myeloma, University of Texas MD Anderson Cancer Center, Houston, TX, USA; UTHealth Houston McGovern Medical School, Houston, TX, USA; Department of Lymphoma/Myeloma, University of Texas MD Anderson Cancer Center, Houston, TX, USA; San Juan Bautista School of Medicine, Caguas, PR, USA; Department of Lymphoma/Myeloma, University of Texas MD Anderson Cancer Center, Houston, TX, USA; UTHealth Houston McGovern Medical School, Houston, TX, USA; Department of Lymphoma/Myeloma, University of Texas MD Anderson Cancer Center, Houston, TX, USA; Division of Pediatrics, University of Texas MD Anderson Cancer Center, Houston, TX, USA; Department of Sarcoma Medical Oncology, University of Texas MD Anderson Cancer Center, Houston, TX, USA; Department of Lymphoma/Myeloma, University of Texas MD Anderson Cancer Center, Houston, TX, USA; UTHealth San Antonio Greehey Children’s Cancer Research Institute, San Antonio, TX, USA; Cook Children’s Hospital, Fort Worth, TX, USA; Division of Pediatrics, University of Texas MD Anderson Cancer Center, Houston, TX, USA; Department of Epidemiology, UTHealth School of Public Health, Houston, TX, USA; Department of Lymphoma/Myeloma, University of Texas MD Anderson Cancer Center, Houston, TX, USA

## Abstract

**Background:**

Neighborhood socioeconomic deprivation has been linked to adverse health outcomes, yet it is unclear whether neighborhood-level social determinants of health (SDOH) measures affect overall survival in adolescent and young adult patients with cancer.

**Methods:**

This study used a diverse cohort of adolescent and young adult patients with cancer (N = 10 261) seen at MD Anderson Cancer Center. Zip codes were linked to Area Deprivation Index (ADI) values, a validated neighborhood-level SDOH measure, with higher ADI values representing worse SDOH.

**Results:**

ADI was statistically significantly worse (*P* < .050) for Black (61.7) and Hispanic (65.3) patients than for White patients (51.2). Analysis of ADI by cancer type showed statistically significant differences, mainly driven by worse ADI in patients with cervical cancer (62.3) than with other cancers. In multivariable models including sex, age at diagnosis, cancer diagnosis, and race and ethnicity, risk of shorter survival for people residing in neighborhoods with the least favorable ADI quartile was greater than for individuals in the most favorable ADI quartile (hazard ratio = 1.09, 95% confidence interval = 1.00 to 1.19, *P* = .043).

**Conclusion:**

Adolescent and young adult patients with cancer and the worst ADI values experienced a nearly 10% increase in risk of dying than patients with more favorable ADI values. This effect was strongest among White adolescent and young adult survivors. Although the magnitude of the effect of ADI on survival was moderate, the presence of a relationship between neighborhood-level SDOH and survival among patients who received care at a tertiary cancer center suggests that ADI is a meaningful predictor of survival. These findings provide intriguing evidence for potential interventions aimed at supporting adolescent and young adult patients with cancer from disadvantaged neighborhoods.

The adolescent and young adult cancer population continues to grow and is expected to surpass 85 000 new cases in 2023 (1). Previous studies have identified survival disparities among adolescent and young adult cancer survivors, specifically noting poorer survival in Hispanic and Black adolescent and young adult patients with cancer than in White patients (2). Other studies have observed disparities in survival when comparing neighborhood type, insurance status, and poverty level (3-6). Though past research has highlighted these disparities in survival within the adolescent and young adult cancer population (7), the underlying sources for these disparities remain unclear.

Social determinants of health (SDOH) are the nonmedical factors that affect health outcomes (8). The adolescent and young adult community faces a unique set of challenges when it comes to SDOH, including difficulty with access to health care, finances, employment, social support, and housing. Thus, nonmedical factors can greatly affect the survival of this population and implicate SDOH as an important variable to study in the context of cancer outcomes in adolescent and young adult patients. Socioeconomic status and health insurance status are among the factors that have been shown to negatively affect overall survival and other outcomes in adolescent and young adult populations with cancer (9,10). The Area Deprivation Index (ADI) is a validated composite score and neighborhood measure of SDOH that incorporates 17 factors that reflect neighborhood housing quality, household characteristics, education quality, income, and employment (8). Recent analyses of ADI as an indicator of SDOH have underscored an independent impact of the local environment on cancer outcomes in diverse populations, even when accounting for the individual SDOH factors (11-13). Though there has been research assessing the impact of individual SDOH components in the setting of adolescent and young adult disparities, studies have yet to use a composite SDOH measure that focuses on the neighborhood environment to investigate cancer outcomes among adolescent and young adult patients.

In this study, we investigated the role of overall neighborhood-level SDOH via the ADI measure in observed survival disparities using a large, racially and ethnically diverse cohort of adolescent and young adult patients with cancer from a single institution. The research conducted in this study was designed to address 3 objectives: 1) to understand neighborhood-level socioeconomic disparities in the adolescent and young adult population, 2) to study the relationship between ADI and overall survival, and 3) to investigate the relationship between ADI and overall survival for different racial and ethnic groups. This project was established to help alleviate the significant knowledge gap regarding the association between neighborhood-level SDOH and disparities in overall survival in the adolescent and young adult patient population.

## Methods

### Study population

Study participants (N = 10 261) included adolescent and young adult patients with cancer diagnosed between the ages of 15 and 39 years and who received treatment at MD Anderson Cancer Center between 2000 and 2016. Patient and tumor characteristics (race and ethnicity, sex, age at diagnosis, date of diagnosis, cancer diagnosis, vital status, date of last follow-up, and zip code at presentation) were obtained from our institutional tumor registry. Race and ethnicity information was collected as Black, White, Hispanic, Asian, American Indian/Alaskan Native, other, or unknown. Due to the limited numbers of individuals in the Asian (n = 33), American Indian/Alaskan Native (n = 16), other (n = 601), or unknown (n = 5) categories, these patients were grouped as “Other”. Individuals with a prior diagnosis of childhood cancer were excluded from this study. This study was approved by the Institutional Review Board of MD Anderson Cancer Center.

### Area Deprivation Index

The ADI from the Public Health Neighborhood Atlas is a composite of 17 SDOH measures (14). Patient zip codes at presentation to MD Anderson Cancer Center were linked to the ADI national ranking values and used for analyses in this study. The ADI is represented as a percentile ranging from 0% to 100%, with the 50% point denoting the “national midpoint.” A low ADI score suggests affluence or prosperity, while a high ADI score signifies elevated levels of deprivation. International patients and patients without zip code information were excluded from analysis.

### Statistical analyses

Student *t* tests or analysis of variance were used to compare ADI values by patient characteristic. Survival was defined as the duration between date of diagnosis and death from any cause or last follow-up, as recorded by the institution’s tumor registry. Cox proportional hazards ratios (HRs) and 95% confidence intervals (CIs) were calculated for survival statistics by ADI mean, median, and quartile. Multivariate analyses were adjusted for sex, age at diagnosis (continuous variable), cancer diagnosis, and race and ethnicity, unless the variable was a stratification factor. Biological (additive) interaction analyses for race and ethnicity and ADI on overall survival were conducted using the method of Andersson et al. (15). Interactions were evaluated through interpretation of the synergy index [SI = (HR_11_ ‒1) / (HR_10_ ‒1) + (HR_01_ ‒1)], with values less than 1 indicative of negative (antagonistic) effects and values greater than 1 indicative of positive (synergistic) effects (15). All other analyses were conducted using Stata, version 17, software (StataCorp, College Station, TX), with a 2-sided *P* = .050 set as the threshold of statistical significance.

## Results

### Patient population

The study population of 10 261 adolescent and young adult patients with cancer was diverse in terms of race and ethnicity, with nearly 40% of the population identifying as non-White race or ethnicity—10.8% Black, 20.8% Hispanic, and 6.4% other races and ethnicities (Table 1). Breast cancer was the most common diagnosis, at 17.6%. The diagnoses with at least 400 patients in the population are shown in Table 1. There were slightly more female patients (55.6%) than male patients (44.4%), and a majority of individuals were diagnosed in the Young Adult age category, defined as individuals 26 to 39 years of age (73.3%). Over a median follow-up of 7.3 years, 4415 deaths were recorded, with more than 80% of the population surviving more than 2 years after diagnosis. Of note, the 5-year survival rate for this population was 65.8%, which is lower than the 85% reported for the adolescent and young adult cancer population nationally, likely because of the high number of patients with relapsed-refractory disease seen at MD Anderson Cancer Center.

**Table 1.** Population characteristics

		Area Deprivation Index quartile			
	Total, No. (%)	Q1 (0-36.8)	Q2 (36.9-56.1)	Q3 (56.2-73.5)	Q4 (73.6-100)
Self-identified race or ethnicity	10 261				
Black	1112 (10.8)	152 (5.9)	256 (10.0)	344 (13.3)	360 (14.1)
Hispanic	2133 (20.9)	261 (10.2)	428 (16.7)	503 (20.0)	941 (36.9)
White	6361 (62.0)	1850 (72.1)	1713 (66.8)	1615 (62.6)	1183 (46.4)
Other	655 (6.4)	303 (11.8)	168 (6.6)	117 (4.5)	67 (2.6)
Cancer type					
Breast	1810 (17.6)	506 (19.7)	438 (17.1)	429 (16.6)	437 (17.1)
Cervical	401 (3.9)	56 (2.2)	85 (3.3)	112 (4.3)	148 (5.8)
Central nervous system	408 (4.0)	114 (4.4)	97 (3.8)	105 (4.1)	92 (3.6)
Colorectal	648 (6.3)	172 (6.7)	168 (6.6)	161 (6.2)	147 (5.8)
Germ-cell tumor	703 (6.9)	138 (5.4)	181 (7.1)	188 (7.30	196 (7.7)
Hodgkin lymphoma	964 (9.4)	243 (9.5)	254 (9.9)	269 (10.4)	198 (7.8)
Leukemia	1370 (13.4)	314 (12.2)	323 (12.6)	344 (13.3)	389 (15.3)
Non-Hodgkin lymphoma	843 (8.2)	212 (8.3)	241 (9.4)	199 (7.7)	191 (7.5)
Sarcoma	1179 (11.5)	314 (12.2)	307 (12.0)	294 (11.4)	264 (10.4)
Other	1935 (18.9)	497 (19.4)	471 (18.4)	478 (18.5)	489 (19.2)
Sex					
Female	5701 (55.6)	1440 (56.1)	1422 (55.4)	1417 (54.9)	1422 (55.7)
Male	4560 (44.4)	1126 (43.9)	1143 (44.6)	1162 (45.1)	1129 (44.3)
Age at diagnosis, median, y	31.5	32	32	31	31
Diagnosis, by age group					
Adolescent: 15-18 y	752 (7.3)	172 (6.7)	191 (7.5)	192 (7.4)	197 (7.7)
Emerging adult: 19-25 y	1984 (19.3)	444 (17.3)	479 (18.7)	544 (21.1)	517 (20.3)
Young adult: 26-39 y	7525 (73.3)	1950 (76.0)	1895 (73.9)	1843 (71.5)	1837 (72.0)
5-y survival rate, %	65.8	67.4	67.3	64.4	64.2
Follow-up time, median (IQR), y^a^	7.3 (2.9-12.2)	7.3 (3.0-11.9)	7.4 (3.1-12.5)	7.0 (2.7-11.9)	7.2 (2.8-12.6)
Vital status					
Alive	5801 (56.5)	1495 (58.4)	1470 (57.5)	1431 (55.8)	1405 (55.4)
Dead	4415 (43.0)	1063 (41.6)	1087 (42.5)	1133 (44.2)	1132 (44.6)

a IQR = intra-quartile range.

### ADI, by patient characteristics

**Figure 1.**
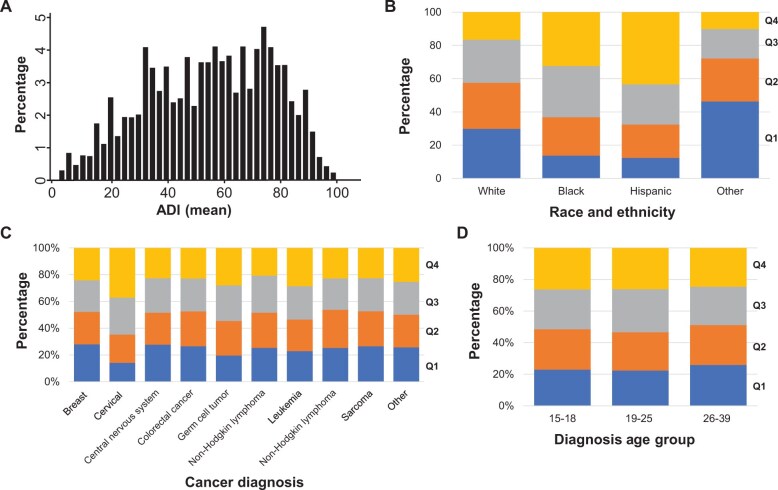
ADI, by patient characteristics. **A)** overall distribution of ADI values in the population. **B)** distribution of ADI quartiles, by race and ethnicity. **C)** distribution of ADI quartiles, by cancer diagnosis. **D)** distribution of ADI quartiles, by diagnosis age group. ADI = Area Deprivation Index; Q = quartile.

Overall, the mean (SD) ADI value for the adolescent and young adult cancer population was 54.7 (22.4) (Figure 1, A). Mean ADI was statistically significantly different by race and ethnicity (*P* < .050), with Hispanic (65.3) and Black (61.7) adolescent and young adult patients living in neighborhoods with worse area deprivation. When assessing the ADI distribution by quartiles (Figure 1, B), Hispanic and Black adolescent and young adult patients with cancer had a higher proportion of patients residing in neighborhoods with the highest levels of deprivation (quartile [Q] 4: 44% and 32%, respectively) compared with White patients and patients of other races and ethnicities (19% and 10%, respectively). Statistically significant differences in mean ADI were observed by cancer diagnosis. Notably, patients diagnosed with cervical cancer had a statistically significantly worse ADI value (62.3) than patients with other cancer diagnoses (pair-wise *P* < .050). Of note, the largest proportion of cancer diagnoses for patients within the Q4 ADI were for cervical cancer (Figure 1, C). ADI was statistically significantly different between diagnosis age groups (*P* < .0100), with the highest mean ADI among patients in the emerging adult category, defined as those diagnosed between 19 and 25 years of age. The distribution of ADI quartiles, however, did not differ between age groups (Figure 1, D). There were no statistically significant differences in ADI by sex or follow-up time category, defined as less than or more than 2 years.

### Survival analysis

**Figure 2.**
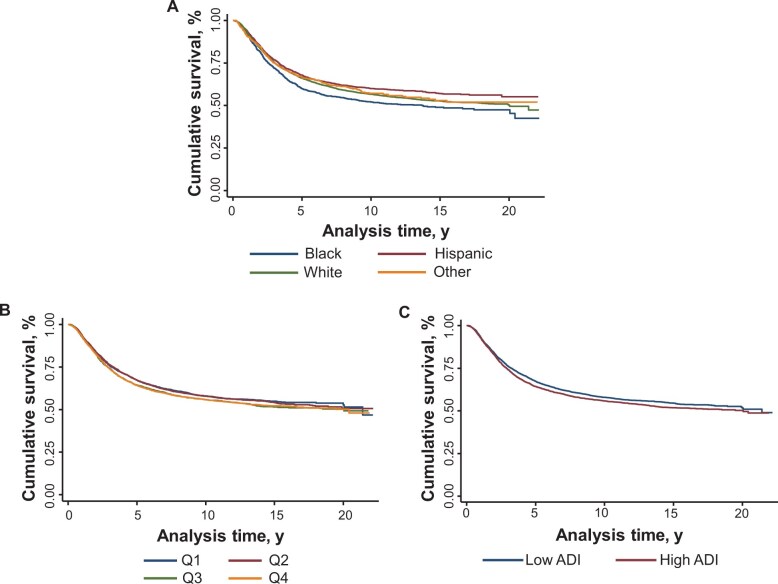
Overall survival in adolescent and young adult cancer survivors. **A)** race and ethnicity. **B)** ADI quartiles. **C)** ADI medians. ADI = Area Deprivation Index.

Hispanic patients experienced favorable survival, and Black patients had worse outcomes compared to other groups (Figure 2, A). When accounting for sex, age at diagnosis, cancer diagnosis, and race and ethnicity, adolescent and young adult patients with cancer residing in the neighborhoods with the highest levels of area deprivation (Q4) had poorer survival than individuals living in areas with the lowest levels (Q1 HR = 1.09, 95% CI = 1.00 to 1.19, *P* = .043) (Table 2 and Figure 2, B). This statistically significant effect is evident in unadjusted and adjusted models for survival when analyzed by ADI as a continuous variable and median (Table 2). The effect sizes for Q3 and Q4 of the ADI were similar to each other, while Q2 did not show a statistically significant increase in hazard ratio compared with Q1. Therefore, we used median ADI categories for subsequent analyses (Figure 2, C).

**Table 2.** Overall survival, by ADI value

	5-y survival rate, %	Log-rank *P*	**Hazard ratio (95% confidence interval)** ^a^	*P*
ADI continuous	65.80		1.002 (1.000 to 1.003)	.011
ADI quartile		.082		
0-25	64.46		(Referent)	
26-49	64.44		1.03 (0.94 to 1.12)	.53
50-74	61.97		1.10 (1.01 to 1.20)	.025
75-100	61.58		1.09 (1.00 to 1.19)	.043
ADI value, median		< .0100		
Favorable	64.45		(Referent)	
Unfavorable	61.77		1.08 (1.02 to 1.15)	.012

a Adjusted for sex, diagnosis age group, cancer type diagnosis, and race and ethnicity. ADI = Area Deprivation Index.

To investigate the potential relationships between ADI and race and ethnicity, stratified analyses were performed. Neighborhood deprivation was a statistically significant factor affecting survival only in White adolescent and young adult patients, with individuals residing in neighborhoods with high deprivation having an 11% increase in risk (HR = 1.11, 95% CI = 1.03 to 1.20, *P* < .0100) compared with those in low deprivation neighborhoods (Table 3). In contrast, ADI was not associated with overall survival in Hispanic patients (HR = 0.97, 95% CI = 0.84 to 1.13, *P* = .73). Among Black patients, the effect size of worse ADI vs favorable ADI (HR = 1.11, 95% CI = 0.93 to 1.33) with regard to survival was similar to that among White patients, but the result was not statistically significant (*P* = .24). This lack of association may be the result of a limited sample size from the Black and Hispanic populations when stratified by ADI categories. In analysis of survival by race and ethnicity, Black race was statistically significantly associated with poor survival in both high and low deprivation neighborhoods, conferring approximately 20% increased risk in both ADI categories (Table 3). No statistically significant interactions between ADI and race and ethnicity affecting overall survival were identified (data not shown), but a suggestive antagonistic interaction between race and ADI for the Hispanic patient population was observed, with a synergy index of 0.20 (95% CI = 0.012 to 3.20) nearing a value below 1.

**Table 3.** Overall survival, by race and ethnicity and ADI category

	5-y survival rate, %	Log-rank *P*	**Hazard ratio (95% confidence interval)** ^a^	*P*
Black only (n = 1103)		.64		
ADI favorable (n = 404)	59.03		(Referent)	
ADI unfavorable (n = 699)	57.02		1.11 (0.93 to 1.33)	.24
Hispanic only (n = 2122)		.81		
ADI favorable (n = 685)	64.63		(Referent)	
ADI unfavorable (n = 1437)	64.96		0.97 (0.84 to 1.13)	.73
White only (n = 6331)		< .0100		
ADI favorable (n = 3553)	65.01		(Referent)	
ADI unfavorable (n = 2778)	61.09		1.11 (1.03 to 1.20)	< .0100
ADI favorable (n = 4642)		.038		
Black (n = 404)	59.03		1.23 (1.05 to 1.43)	< .0100
Hispanic (n = 685)	64.63		1.04 (0.91 to 1.19)	.55
White (n = 3553)	65.01		(Referent)	
ADI unfavorable (n = 4914)		< .00100		
Black (n = 699)	57.02		1.19 (1.06 to 1.35)	< .0100
Hispanic (n = 1437)	64.96		0.94 (0.85 to 1.04)	.23
White (n = 2778)	61.09		(Referent)	

a Adjusted for sex, diagnosis age group, and cancer type diagnosis. ADI = Area Deprivation Index.

## Discussion

In this study, we evaluated a large (N = 10 261) and diverse cohort of adolescent and young adults with cancer from a single institution to explore the association between neighborhood-level SDOH and overall survival. Adolescent and young adult patients with cancer residing in areas with higher neighborhood-level deprivation experienced a nearly 10% increased risk of dying than those in lower-deprivation areas, implicating SDOH as valuable prognostic factor affecting long-term survival. The potential implications of this finding are concerning because this effect was observed in adolescent and young adult patients with cancer at a single comprehensive cancer center that is biased towards individuals who are insured.

Past studies that have used ADI as a measure of SDOH have found inverse relationships between deprivation levels and survival that are aligned with our findings (16,17). A study focusing on disparities among patients diagnosed with primary central nervous system lymphoma made the same conclusion concerning the impact of worse ADI on overall survival. Primary central nervous system lymphoma is different from the cancers included in this analysis in that it is typically a disease of older adults and has a poor prognosis, yet the similarity of the relationship between ADI and survival is interesting. Another study in adult breast, prostate, lung, and colorectal cancers described similar relationships between SDOH and overall survival, even when accounting for individual-level socioeconomic status (18). This study used Surveillance, Epidemiology, and End Results data to gather patient information, indicating that the patients obtained their medical care across the country rather than at a single institution, as in this study. In addition, their population consisted of older adult patients, which differs from the population of the current study (18,19). Although past study populations have different features from the current adolescent and young adult cancer population, it provides compelling evidence that research using ADI is needed within other populations—particularly among individuals that are underinsured or uninsured. Thus, our analysis is the first to extend the effect of SDOH to adolescent and young adult patients with cancer treated in a single institution and further supports the importance of neighborhood-level deprivation as a potential driver of the persistent survival disparities observed in this community.

Racial disparities in overall survival among adolescent and young adult patients with cancer have been reported previously (20,21). In an analysis of more than 80 000 adolescent and young adult patients with cancer from Texas, Black men and women had poorer 5-year survival rates than White individuals diagnosed with the same cancer (7). It is thought that structural racism within health care may contribute to these disparities (22-26). Studies have linked structural racism to poor physical and mental health outcomes, with studies also identifying differences in time to treatment and treatments offered to Black patients that would potentially contribute to differences in survival (27-32). Black adolescent and young adult patients with cancer in our cohort also experienced worse survival than White and Hispanic patients. This effect was consistent when stratified by ADI, suggesting that the survival disadvantage among Black adolescent and young adults with cancer is independent of their neighborhood environment. It is worth noting, however, that the current study participants were all treated at a single academic cancer center, which could limit generalizability to other cancer care settings. Furthermore, the effects of structural racism that affect access and receipt of care are often difficult to measure. More investigation is needed in this area that incorporates detailed individual-level data, such as time to treatment, disease stage at treatment, and frequency of follow-up care, to fully explore this relationship.

Conversely, the Hispanic adolescent and young adult cancer population had a more favorable survival rate than other adolescent and young adult patients with cancer, and this effect was not affected by ADI. The Hispanic paradox, or what some call the “barrio-advantage,” may contribute to the improved survival observed in our study, regardless of local neighborhood disadvantage (33,34). This paradox describes improved outcomes among Hispanic vs non-Hispanic communities thought to be a result of strong social networks and support. Although not significant, we did observe a suggestively, slightly antagonistic interaction between race and ethnicity and ADI in the analysis of our Hispanic patients compared with White adolescent and young adult patients with cancer. This finding is in line with the Hispanic paradox hypothesis, where strong social support and other potentially beneficial cultural factors associated with Hispanic communities attenuate the adverse effect of neighborhood socioeconomic deprivation. More research on the impact of intricate social networks, possibly through assessment of neighborhood social capital and other measures of network support, in Hispanic adolescent and young adult cancer populations may provide more context for our results with respect to this phenomenon.

Worse ADI representing poorer neighborhood environment was associated with a survival disadvantage among White adolescent and young adults with cancer, with an 11% increase in death among individuals living in neighborhoods with a worse ADI. Although White people make up the majority of the total number of people living in poverty within the United States, disparities between high-income and low-income Whites patients are far less well studied than those disparities for racial and ethnic groups (35). Our results provide evidence that ADI may contribute to disparities within the White community, though more research is needed in this setting. This finding also suggests a multifaceted relationship among ADI, race and ethnicity, and overall survival.

Adolescent and young adult patients diagnosed with cervical cancer resided in neighborhoods with statistically significantly worse neighborhood-level deprivation compared with other cancer diagnoses. Underlying disparities in cervical cancer screening and prevention may be the driver of this result. Research evaluating human papillomavirus (HPV) vaccination by geographic measures in the adolescent and young adult population has concluded that people’s local area can affect vaccination rates, suggesting an increase in cervical cancer incidence and reinforcing our findings in the present cervical cancer population (36,37). Despite the presence of HPV vaccinations as a method of cervical cancer prevention that became available to the public in 2006 (38,39), disparities in vaccination uptake continue (40,41). Furthermore, current vaccine recommendations are for the nonavalent formulation of the HPV vaccine, which was not approved until 2014 (42). At the end of the cohort diagnosis period (2000-2016), the HPV vaccination rate in the United States was only about 43% for the completed HPV series and 60% for greater than or equal to 1 vaccine dose (43). In addition, cervical cancer screening rates are affected by county-level vulnerability and area deprivation level, either because of socioeconomic issues or geographical inaccessibility (44,45). Another study using ADI found higher deprivation levels associated with decreased cervical cancer screening, lending support to our own conclusions (45). Differences in health literacy may also be a contributing factor. In Texas, health literacy has been shown to be a barrier in the Hispanic and Black community (46,47). Interventions directed at disadvantaged groups have supported the importance of group-specific education materials and their ability to increase health-care knowledge and perceptions (48). The timeline of HPV vaccine approval, vaccination rates, general vaccine hesitancy, and health literacy may have all influenced the results we saw in this cohort (49-51).

This study has several strengths, including long follow-up to enable survival assessment for a population that is characterized by favorable prognosis overall. The large sample size and the diversity of the population in terms of race and ethnicity, ADI, and cancer diagnoses enabled us to conduct robust stratified and interaction analyses. Information regarding disease stage at presentation would be beneficial for exploring whether ADI is linked to delays in treatment and higher disease stage at presentation, both factors that would directly affect survival. Detailed treatment information would also enhance the robustness of this analysis, and efforts to collect these data are ongoing to enable cancer type–specific analyses. The single-center cohort of patients treated at MD Anderson Cancer Center is a strength in that it minimizes concerns regarding the effect of access to care: All participants included in this analysis received best practices–driven care, reducing heterogeneity in the treatments received. The preexisting barriers patients face to gain access to care at MD Anderson Cancer Center, a tertiary cancer center, such as lack of health-care access, financial means, transportation, and geographical location, may introduce a sampling bias and reduce the transferability of our findings to other adolescent and young adult cancer populations. Based on these barriers, our cohort would be expected to have worse ADI values than the general population or individuals receiving care at community hospitals. For patients unable to obtain care at MD Anderson Cancer Center, they may have less access to highly specialized cancer care and novel clinical trials. These barriers may have attenuated some of our results because the prognostic features of ADI were identified among a population with relatively high SDOH. Future studies in adolescent and young adult cancer populations with lower socioeconomic status and patients seen in other care-delivery settings would be beneficial to fully establish the prognostic ability of ADI. We would expect a larger magnitude of effect of the inverse relationship between ADI and survival when studied in the broader adolescent and young adult population.

The composite ADI values used in the analysis were generated by patient zip codes. The exact addresses would have allowed for census block–based ADI by geocoding and enable investigation of other SDOH measures beyond that captured by ADI. Future studies with robust longitudinal residential information would be of interest to assess geographical changes between the time of diagnosis and time of censoring study participants. This consideration is important for the adolescent and young adult population because of their increased mobility during young adulthood and the potential impact of these transitions on ADI values.

In conclusion, this study demonstrated the effect of ADI on overall survival in the context of a racially and ethnic diverse cohort to address survival disparities within the adolescent and young adult cancer population. Overall, our findings implicate ADI as an important prognostic factor in adolescent and young adult cancer survival. This information warrants continued investigation to better explain the impact of ADI on the adolescent and young adult population. With more investigation, ADI may guide individualized social interventions if it proves useful as a screening tool. Overall, the intriguing findings underscore the need for continued support in disadvantaged populations.

## Data Availability

Deidentified data may be made available upon reasonable request to the corresponding author.

## References

[1] *Surveillance, Epidemiology, and End Results (SEER) Program*. https://seer.cancer.gov/statfacts/html/aya.html. Accessed June 26, 2023.

[2] Keegan TH , DeRouen MC, Parsons HM, et al Impact of treatment and insurance on socioeconomic disparities in survival after adolescent and young adult Hodgkin lymphoma: a population-based study. Cancer Epidemiol Biomarkers Prev. 2016;25(2):264-273. 26826029 10.1158/1055-9965.EPI-15-0756 PMC4767568

[3] Berkman AM , Andersen CR, Hildebrandt MAT, et al Risk of early death in adolescents and young adults with cancer: a population-based study. J Natl Cancer Inst. 2023;115(4):447-455. 36682385 10.1093/jnci/djac206 PMC10086632

[4] Berkman AM , Andersen CR, Roth ME, et al Cardiovascular disease in adolescent and young adult cancer survivors: Impact of sociodemographic and modifiable risk factors. Cancer. 2023;129(3):450-460. 36464957 10.1002/cncr.34505 PMC11840550

[5] Berkman AM , Mittal N, Roth ME. Adolescent and young adult cancers: unmet needs and closing the gaps. Current Opinion in Pediatrics. 2023;35(1):84-90. 36592027 10.1097/MOP.0000000000001200 PMC12859744

[6] Kahn JM , Beauchemin M. Improving health equity and reducing disparities in pediatric and adolescent/young adult oncology: in support of clinical practice guidelines. J Natl Compr Cancer Netw. 2021;19(6):765-769. 10.6004/jnccn.2021.7048 34214972

[7] Murphy CC , Lupo PJ, Roth ME, et al Disparities in cancer survival among adolescents and young adults: a population-based study of 88 000 patients. JNCI-J Natl Cancer Inst. 2021;113(8):1074-1083. 10.1093/jnci/djab006 PMC8328976 33484568

[8] Hacker K , Auerbach J, Ikeda R, et al Social determinants of health-an approach taken at CDC. J Public Health Manage Pract. 2022;28(6):589-594. 10.1097/PHH.0000000000001626 PMC9555578 36194813

[9] Ryder-Burbidge C , Diaz RL, Barr RD, et al The burden of late effects and related risk factors in adolescent and young adult cancer survivors: a scoping review. Cancers. 2021;13(19):4870. 10.3390/cancers13194870 PMC8508204 34638350

[10] Semrad TJ , Li Q, Goldfarb M, et al Influence of socioeconomic status on incident medical conditions in two-year survivors of adolescent or young adult differentiated thyroid cancer. J Adolesc Young Adult Oncol. 2021;10(5):521-533. 33512275 10.1089/jayao.2020.0142 PMC8666796

[11] Shen JF , Bernard F, Sheppard VB, et al Neighborhood disadvantage and biological aging biomarkers among breast cancer patients. Sci Rep. 2022;12(1):11006. 35773311 10.1038/s41598-022-15260-0 PMC9246873

[12] Ohlsen TJD , Doody DR, Mueller BA, et al Population-based impact of rurality and neighborhood-level socioeconomic disadvantage on pediatric cancer mortality in Washington State. Cancer Epidemiol Biomarkers Prev. 2023;32(1):141-148. 36343539 10.1158/1055-9965.EPI-22-0897 PMC9839485

[13] Luningham JM , Seth G, Saini G, et al Association of Race and Area Deprivation With Breast Cancer Survival Among Black and White Women in the State of Georgia. Jama Network Open. 2022;5(10):e2238183. 36306134 10.1001/jamanetworkopen.2022.38183 PMC9617173

[14] Kind AJH , Buckingham WR. Making neighborhood-disadvantage metrics accessible—the neighborhood atlas. New Engl J Med. 2018;378(26):2456-2458. 29949490 10.1056/NEJMp1802313 PMC6051533

[15] Andersson T , Alfredsson L, Kallberg H, et al Calculating measures of biological interaction. Eur J Epidemiol. 2005;20(7):575-579. 16119429 10.1007/s10654-005-7835-x

[16] Deng XY , Yang XG, Yang CL, et al Socioeconomic deprivation and survival outcomes in primary central nervous system lymphomas. Front Oncol. 2022;12:929585. 36091170 10.3389/fonc.2022.929585 PMC9459230

[17] Kim T. Relationship of neighborhood and individual socioeconomic status on mortality among older adults: evidence from cross-level interaction analyses. PLoS One. 2022;17(5):e0267542. 35588127 10.1371/journal.pone.0267542 PMC9119539

[18] Cheng E , Soulos PR, Irwin ML, et al Neighborhood and individual socioeconomic disadvantage and survival among patients with nonmetastatic common cancers. JAMA Network Open. 2021;4(12):e2139593. 34919133 10.1001/jamanetworkopen.2021.39593 PMC8683967

[19] Akinyemiju TF , Soliman AS, Johnson NJ, et al Individual and neighborhood socioeconomic status and healthcare resources in relation to black-white breast cancer survival disparities. J Cancer Epidemiol. 2013;2013:490472. 23509460 10.1155/2013/490472 PMC3590635

[20] Berkman AM , Andersen CR, Puthenpura V, et al Impact of race, ethnicity, and socioeconomic status over time on the long-term survival of adolescent and young adult Hodgkin lymphoma survivors. Cancer Epidemiol Biomarkers Prev. 2021;30(9):1717-1725. 34244160 10.1158/1055-9965.EPI-21-0103 PMC8419153

[21] DeRouen MC , Parsons HM, Kent EE, et al Sociodemographic disparities in survival for adolescents and young adults with cancer differ by health insurance status. Cancer Causes Control. 2017;28(8):841-851. 28660357 10.1007/s10552-017-0914-y PMC5572560

[22] Williams DR , Lawrence JA, Davis BA. Racism and health: evidence and needed research. Annu Rev Public Health. 2019;40:105-125. 30601726 10.1146/annurev-publhealth-040218-043750 PMC6532402

[23] Stiles-Shields C , Cummings C, Montague E, et al A call to action: using and extending human-centered design methodologies to improve mental and behavioral health equity. Front Digit Health. 2022;4:848052. 35547091 10.3389/fdgth.2022.848052 PMC9081673

[24] Moke DJ , Tsai K, Hamilton AS, et al Emerging cancer survival trends, disparities, and priorities in adolescents and young adults: a California cancer registry-based study. JNCI Cancer Spectr. 2019;3(2):pkz031. 31276099 10.1093/jncics/pkz031 PMC6597054

[25] Avila JC , Livingston JA, Rodriguez AM, et al Disparities in adolescent and young adult sarcoma survival: analyses of the Texas cancer registry and the national SEER data. J Adolesc Young Adult Oncol. 2018;7(6):681-687. 30096005 10.1089/jayao.2018.0034 PMC6909766

[26] Keegan THM , Grogan RH, Parsons HM, et al Sociodemographic disparities in differentiated thyroid cancer survival among adolescents and young adults in California. Thyroid. 2015;25(6):635-648. 25778795 10.1089/thy.2015.0021 PMC4490589

[27] Nogueira LM , Sineshaw HM, Jemal A, et al Association of race with receipt of proton beam therapy for patients with newly diagnosed cancer in the US, 2004-2018. JAMA Netw Open. 2022;5(4):e228970. 35471569 10.1001/jamanetworkopen.2022.8970 PMC9044116

[28] Schlottmann F , Gaber C, Strassle PD, et al Disparities in esophageal cancer: less treatment, less surgical resection, and poorer survival in disadvantaged patients. Dis Esophagus. 2020;33(2):doz045. 10.1093/dote/doz045 PMC8205620 31076759

[29] Blom EF , ten Haaf K, Arenberg DA, et al Disparities in receiving guideline-concordant treatment for lung cancer in the United States. Ann Am Thorac Soc. 2020;17(2):186-194. 31672025 10.1513/AnnalsATS.201901-094OC PMC6993802

[30] Sukniam K , Kasbi AA, Ali M, et al Disparities in time to treatment for breast cancer. Anticancer Res. 2022;42(12):5813-5818. 36456136 10.21873/anticanres.16088

[31] Cox SR , Daniel CL. Racial and ethnic disparities in laryngeal cancer care. J Racial Ethn Health Disparities. 2022;9(3):800-811. 33733426 10.1007/s40615-021-01018-3

[32] Lillard JW , Moses KA, Mahal BA, et al Racial disparities in Black men with prostate cancer: a literature review. Cancer. 2022;128(21):3787-3795. 36066378 10.1002/cncr.34433 PMC9826514

[33] Keegan THM , Quach T, Shema S, et al The influence of nativity and neighborhoods on breast cancer stage at diagnosis and survival among California Hispanic women. BMC Cancer. 2010;10:603. 21050464 10.1186/1471-2407-10-603 PMC2988754

[34] Boen CE , Hummer RA. Longer-but harder-lives?: the Hispanic health paradox and the social determinants of racial, ethnic, and immigrant-native health disparities from midlife through late life. J Health Soc Behav. 2019;60(4):434-452. 31771347 10.1177/0022146519884538 PMC7245019

[35] Bowie JV , Juon HS, Dubay LC, et al Cancer prevention behaviors in low-income urban whites: an understudied problem. J Urban Health-Bull NY Acad Med. 2009;86(6):861-871. 10.1007/s11524-009-9391-2 PMC2791816 19597995

[36] Do EK , Rossi B, Miller CA, et al Area-level variation and human papillomavirus vaccination among adolescents and young adults in the united states: a systematic review. Cancer Epidemiol Biomarkers Prev. 2021;30(1):13-21. 33008874 10.1158/1055-9965.EPI-20-0617 PMC8108385

[37] Pruitt SL , Schootman M. Geographic disparity, area poverty, and human papillomavirus vaccination. Am J Prev Med. 2010;38(5):525-533. 20409501 10.1016/j.amepre.2010.01.018 PMC3259737

[38] Harper DM , Franco EL, Wheeler CM, et al; HPV Vaccine Study Group. Sustained efficacy up to 4.5 years of a bivalent L1 virus-like particle vaccine against human papillomavirus types 16 and 18: Follow-up from a randomised control trial. Lancet. 2006;367(9518):1247-1255. 16631880 10.1016/S0140-6736(06)68439-0

[39] Villa LL , Costa RL, Petta CA, et al Prophylactic quadrivalent human papillomavirus (types 6, 11, 16, and 18) L1 virus-like particle vaccine in young women: a randomised double-blind placebo-controlled multicentre phase II efficacy trial. Lancet Oncol. 2005;6(5):271-278. 15863374 10.1016/S1470-2045(05)70101-7

[40] Hirth J. Disparities in HPV vaccination rates and HPV prevalence in the United States: a review of the literature. Hum Vaccin Immunother. 2019;15(1):146-155. 30148974 10.1080/21645515.2018.1512453 PMC6363146

[41] Hirth J , McGrath CJ, Kuo YF, et al Impact of human papillomavirus vaccination on racial/ethnic disparities in vaccine-type human papillomavirus prevalence among 14-26 year old females in the U.S. Vaccine. 2018;36(50):7682-7688. 30377066 10.1016/j.vaccine.2018.10.075 PMC6289515

[42] Markowitz LE , Dunne EE, Saraiya M, et al; Centers for Disease Control and Prevention (CDC). Human papillomavirus vaccination recommendations of the Advisory Committee on Immunization Practices (ACIP). MMWR Recomm Rep. 2014;63(RR-05):1-30. 25167164

[43] Walker TY , Elam-Evans LD, Singleton JA, et al National, regional, state, and selected local area vaccination coverage among adolescents aged 13-17 years—United States, 2016. MMWR-Morb Mort Weekly Rep. 2017;66(33):874-882. 10.15585/mmwr.mm6633a2 PMC5687818 28837546

[44] Bauer C , Zhang KH, Xiao Q, et al County-level social vulnerability and breast, cervical, and colorectal cancer screening rates in the US, 2018. JAMA Netw Open. 2022;5(9):e2233429. 36166230 10.1001/jamanetworkopen.2022.33429 PMC9516325

[45] Kurani SS , McCoy RG, Lampman MA, et al Association of neighborhood measures of social determinants of health with breast, cervical, and colorectal cancer screening rates in the US Midwest. JAMA Network Open. 2020;3(3):e200618. 32150271 10.1001/jamanetworkopen.2020.0618 PMC7063513

[46] Akinlotan M , Bolin JN, Helduser J, et al Cervical cancer screening barriers and risk factor knowledge among uninsured women. J Commun Health. 2017;42(4):770-778. 10.1007/s10900-017-0316-9 PMC5494033 28155005

[47] Fernandez ME , Savas LS, Lipizzi E, et al Cervical cancer control for Hispanic women in Texas: strategies from research and practice. Gynecol Oncol 2014;132(suppl 1(01)):S26-S32. 24398135 10.1016/j.ygyno.2013.12.038 PMC4053183

[48] Casillas JN , Schwartz LF, Gildner JL, et al Engaging Latino Adolescent and Young Adult (AYA) cancer survivors in their care: piloting a photonovela intervention. J Cancer Educ. 2021;36(5):971-980. 32333369 10.1007/s13187-020-01724-2 PMC10132777

[49] Morales-Campos DY , Zimet GD, Kahn JA. Human papillomavirus vaccine hesitancy in the United States. Pediatric Clin North Am. 2023;70(2):211-226. 10.1016/j.pcl.2022.11.002 36841591

[50] Victory M , Do TQN, Kuo YF, et al Parental knowledge gaps and barriers for children receiving human papillomavirus vaccine in the Rio Grande Valley of Texas. Hum Vaccin Immunother. 2019;15(7-8):1678-1687. 31170031 10.1080/21645515.2019.1628551 PMC6746477

[51] Kim S , Zhou K, Parker S, et al Perceived barriers and use of evidence-based practices for adolescent HPV vaccination among east Texas providers. Vaccines. 2023;11(4):728. 10.3390/vaccines11040728 PMC10146224 37112640

