# Correction to: Neighborhood-level social determinants of health burden among adolescent and young adult cancer patients and impact on overall survival

**DOI:** 10.1093/jncics/pkaf063

**Published:** 2025-06-20

This is a correction to: Elizabeth R Rodriguez, Tori Tonn, Midhat Jafry, Sairah Ahmed, Branko Cuglievan, J Andrew Livingston, Christopher R Flowers, Gregory J Aune, Karen H Albritton, Michael E Roth, Qian Xiao, Michelle A T Hildebrandt, Neighborhood-level social determinants of health burden among adolescent and young adult cancer patients and impact on overall survival, *JNCI Cancer Spectrum*, Volume 8, Issue 4, August 2024, https://doi.org/10.1093/jncics/pkae062

The Funding statement has been updated in the original published article to acknowledge the following additionally: “Christopher R. Flowers received support from the Cancer Prevention and Research Institute of Texas (RR190079), where he is a Cancer Prevention and Research Institute of Texas Scholar in Cancer Research.”

